# Filament Geometry Induced Bipolar, Complementary, and Unipolar Resistive Switching under the Same Set Current Compliance in Pt/SiO_x_/TiN

**DOI:** 10.1038/srep15374

**Published:** 2015-10-22

**Authors:** Dong-Hyeok Lim, Ga-Yeon Kim, Jin-Ho Song, Kwang-Sik Jeong, Dae-Hong Ko, Mann-Ho Cho

**Affiliations:** 1 Department of Physics, Yonsei University, Seoul 120-749, Korea; 2 Department of Material Science and Engineering, Yonsei University, Seoul 120-749, Korea

## Abstract

The decidedly unusual co-occurrence of bipolar, complementary, and unipolar resistive switching (BRS, CRS, and URS, respectively) behavior under the same high set current compliance (set-CC) is discussed on the basis of filament geometry in a Pt/SiO_x_/TiN stack. Set-CC-dependent scaling behavior with relations I_reset_ ~ R_0_^–α^ and V_reset_ ~ R_0_^–β^ differentiates BRS under low set-CC from other switching behaviors under high set-CC due to a low α and β involving a narrow filamentary path. Because such co-occurrence is observed only in the case of a high *α* and β involving a wide filamentary path, such a path can be classified into three different geometries according to switching behavior in detail. From the cyclic switching and a model simulation, we conclude that the reset of BRS originates from a narrower filamentary path near the top electrode than that of CRS due to the randomness of field-driven migration even under the same set-CC. Also, we conclude that URS originates from much narrower inversed conical filamentary path. Therefore, filament-geometry-dependent electric field and/or thermal effects can precisely describe the entire switching behaviors in this experiment.

Resistive-switching random access memory (RRAM) has attracted considerable interest as a new, non-volatile, next-generation memory due to its superior properties, which include its simple structure, high density, high speed, and a variety of functionalities such as bipolar, complementary, and unipolar resistive switching (BRS, CRS, and URS, respectively)^1^,^2^,^3^. It has been acknowledged that RRAMs can be roughly grouped into cation-based, anion-based, and electronic systems^4^,^5^. Between the cation- and anion-based systems, the former are frequently referred to as a programmable metallization cell (PMC) or a conducting-bridge RAM (CBRAM), which is the closest to commercialization by virtue of its superior overall performance^6^. However, the latter, which is referred to as valence change memory continues to be a subject of investigation because of the potential for utilizing the fundamental properties of electrons in defect-engineered oxides^7^,^8^. In contrast to the cation-based memory, which could generate metal chains that would involve a Joule heating effect to disrupt the metal conduction channel during the reset process, the anion-based version would exhibit various and complicated switching behavior^4^,^5^. Separating out the diverse switching processes from one another could possibly increase performance limits with regard to reproducibility and power consumption^2^,^5^. A Pt-dispersed silicon oxide system as an anion-based or electronic memory has recently been reported to improve device performance^2^,^9^. However, it is difficult to improve overall device performance due to the trade-off between performance factors^2^. Since the diverse switching processes are related to differences in the formation and deformation of a conduction path, detailed investigations are required to determine the relations between switching behavior and the evolution of a conduction path.

Regardless of the two distinctive switching types, the electric field effect and/or the thermal effect can be inevitably related to the switching mechanisms due to the conventional voltage-driven device operation scheme and the high current on-state *via* the conduction path^5^,^10^. In the case of anionic systems, it is understood that BRS and URS are field effect and thermal effect dominating processes, respectively^4^,^5^,^11^. In addition, although electrical characterization was first reported in a device consisting of two anti-serially connecting anionic BRS elements and the term ‘CRS’ was then used in a device consisting of two anti-serially connecting cationic BRS elements, CRS has been observed, even in a single layered anionic system^3^,^12^,^13^. In the single layered system, the alternating formation of inversed conduction path can generate CRS depending on the sign of the voltage, due to the limited amount of ionic sources^13^,^14^,^15^. Therefore, the appearance of CRS added to BRS and URS in a single layered oxide requires a more precise understanding within the field and/or the thermal effects.

It is noteworthy that the silicon-oxide-based resistive switching is distinguished from other metal-oxide-based ones for several reasons. In practice, the metal-dispersed system is meaningful for silicon oxide, because metallic sources are not desirable for use in metal-oxide-based resistive switching due to their original disadvantage of having an immoderate low resistive conduction path. A typical result is the threshold atomic composition of oxygen in silicon oxide: i.e., normal (V_reset_ < V_set_) URS is exhibited below the threshold, while abnormal (V_reset_ > V_set_) URS occurs over the threshold due to the formation of different defects^8^. Another result is Si-Si nano-crystalline formation as a conduction path in silicon oxide^16^,^17^.

In addition to these results, our experimental results for the Pt/SiO_x_/TiN structure showed interesting behavior, in that BRS, CRS, and URS were observed, even for the same set current compliance (set-CC). The unusual switching behavior, which is in contrast to reported data for a metal-oxide-based system, permits a more concrete understanding of the switching mechanism to be developed. Moreover the dependence of the conduction path on the switching behavior clearly showed the significance of conduction path geometry, as opposed to the current level. Thus, we conclude that our experimental results represent an example for developing the argument that Si-NCs could function as a filamentary path in SiO_x_-based RRAM. The reason for this is because significantly different Si-NCs-mediated RRAM which is distinguished from oxygen-vacancy-mediated one has not been reported yet. A direct comparison between Si-NCs and oxygen vacancies in the same device is more plausible but less feasible. A variety of Si-NCs-mediated experimental results should be collected and then generalized due to the infeasibility. Therefore, the novelty of this work can be found in the observation of unusual switching behavior which is possibly correlated with the formation of Si-NCs beyond the merely simple observation of Si-NCs.

## Methods

In this work, a 100-nm-thick TiN bottom electrode (BE) was deposited on a SiO_2_/Si substrate at room temperature *via* DC sputtering. A 10-nm-thick SiO_x_ layer was deposited on a TiN BE at room temperature using ion beam sputter deposition (IBSD). A high-purity Si target was pre-sputtered for 30 min in order to remove the oxidized surface and contamination on the Si target. The base and working pressures for the IBSD were 2.0 × 10^−8^ and 7.0 × 10^−5^ Torr, respectively. The atomic composition ratio between Si and O was confirmed by using X-ray photoelectron spectroscopy (XPS), where the Si:O ratio was determined to be about 1:1.9 (x = 1.9, data not shown here) in the pristine SiO_x_. Square patterns (10 μm by 10 μm) were developed using a photo mask on the SiO_x_ at room temperature and 100-nm-thick Pt electrodes deposited by DC sputtering were fabricated by means of a lift-off method. The resistive switching (RS) characteristics were measured by applying a voltage to the Pt electrode and grounding the TiN electrode. Using a semiconductor characterization analyzer (Agilent B1500A) the device was activated by applying a positive forming bias with a current compliance of 500 μA in both the voltage-sweep-inducing and voltage-pulse-inducing methods. Bipolar switching was observed when the voltage sweep scheme of ±2 V was applied with various current compliances ranging from 500 μA to 7 mA. Voltages of 0 to +3 V were applied for the set and 0 to +1.8 V for the reset in unipolar switching. The set pulses for the voltage-pulse scheme (height/width) were −3 V/25 ns or −5 V/35 ns and the reset pulses were +1.5 V/(0.1 ms to 2.5 ms), +2.3 V/50 μs, or +2.5 V/25 μs. The incorporation of oxygen vacancies and the formation of Si crystallites were confirmed *via* X-ray absorption spectroscopy (XAS) and transmission electron microscopy (TEM), respectively.

## Results and Discussion

**Figure 1:**
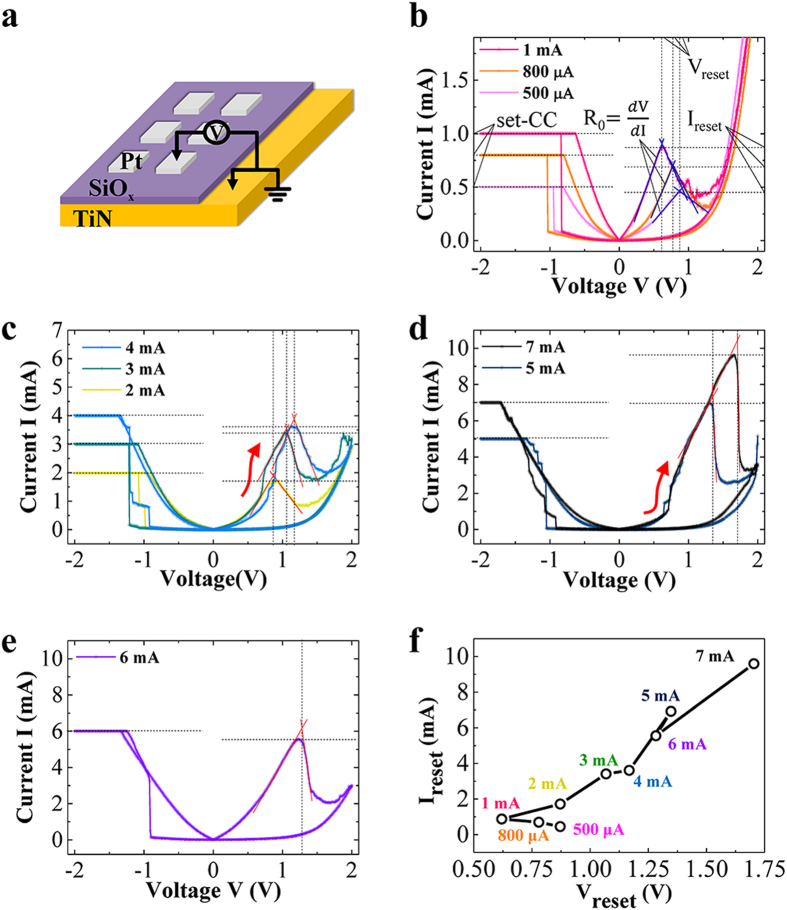
Device structure and electrical characterizations depending on a set current compliance (set-CC). (**a**) A schematic diagram of the device structure and electrical characterization setup. (**b**) The V_reset_ decreases under the increasing set-CC from 500 μA to 1 mA. (**c**) In contrast, the V_reset_ increases under the set-CC of 2–4 mA. A CRS-like I-V characteristic appears. (red arrow) (**d**) The V_reset_ increases under the set-CC of 5–7 mA except 6 mA. (**e**) The CRS-like I-V characteristic disappears under the set-CC of 6 mA. (**f**) Correlation between V_reset_ and I_reset_ is represented.

We investigated the switching characteristics of the Pt/SiO_x_/TiN structure under various set-CC settings. A schematic diagram of the set up used for the measurements is shown in Fig. 1a. In this setup, Pt was biased while TiN was grounded. A sufficiently large top electrode was used to avoid thermal damage to the electrode, as described in the experimental section. The measured current density is lower than the well-known value of 10^6^ A/cm^2^ inducing a significant Joule heating effect in a typical metal oxide^5^. However, a Joule heating effect can actually arise to some degree because the possibly narrower conduction path than the electrode size increases resistivity. Therefore, the thermal effects play a role in the switching process according to the geometry and the material of conduction path.

In order to investigate the effects caused by the switching process, the positive voltage for the switching of the device was swept with a current compliance of 500 μA (or 5 × 10^3^ A/cm^2^ in current density) as an electroforming process for all samples. A negative voltage sweep then conducted as the set-operation under the set-CC from 500 μA (5 × 10^3^ A/cm^2^) to 7 mA (7 × 10^4^ A/cm^2^). The reset-operation was then completed after a positive voltage sweep without a reset-CC. Figure 1b-e shows sequential switching as a function of the stepwise increased set-CC. According to the switching behavior, the sequential switching can be classified into four types, as shown in Fig. 1b–e. Under the set-CC region of 500 μA − 1 mA, the V_reset_ defined as the voltage at which the current starts to drop gradually decreased with the set-CC, as shown in the vertical dashed lines in Fig. 1b. In contrast, the V_reset_ increased with increasing set-CC from 2 mA to 4 mA, as shown in Fig. 1c. Interestingly, an additional current jump before the V_reset_ indicated by the red arrow in Fig. 1c was observed, which is very similar to CRS behavior. The relation between the set-CC and V_reset_ shows a contrasting tendency for high or low set-CC with respect to 1 mA. This result is comparable to the scaling behavior in URS, because the relation between V_reset_ (or I_reset_) and the resistance R_0_ reflects the geometry of the conduction path. Considering the geometry of the filamentary conduction path into either a multiple (equivalently wide) or single (equivalently narrow) connection to the electrode as the scaling theory, the formation and deformation of the conduction path can be qualitatively compared^18^,^19^. At a set-CC of 5 mA, CRS behavior with a larger I_reset_ than the set-CC is induced, as shown in Fig. 1d. However, when the set-CC was increased to 6 mA, CRS behavior is not generated and the I_reset_ was lower than the set-CC in Fig. 1e. When the set-CC reached 7 mA, the switching behavior then returned back to CRS behavior with a higher I_reset_ than the set-CC. The switching behavior of the Pt/SiO_x_/TiN structure under various set-CC settings is summarized in Fig. 1f. The relation between I_reset_ and V_reset_ shows a contrasting tendency near 1 mA set-CC conditions. In addition, a distinct switching behavior occurs at 6 mA set-CC conditions. The former indicates that the I_reset_ or V_reset_ with the resistance R_0_ of the conduction path is controlled by the set-CC condition, which, in turn, is related to the geometry of the conduction path, while the latter implies that an additional effect exists, which is not controllable by the set-CC.

**Figure 2:**
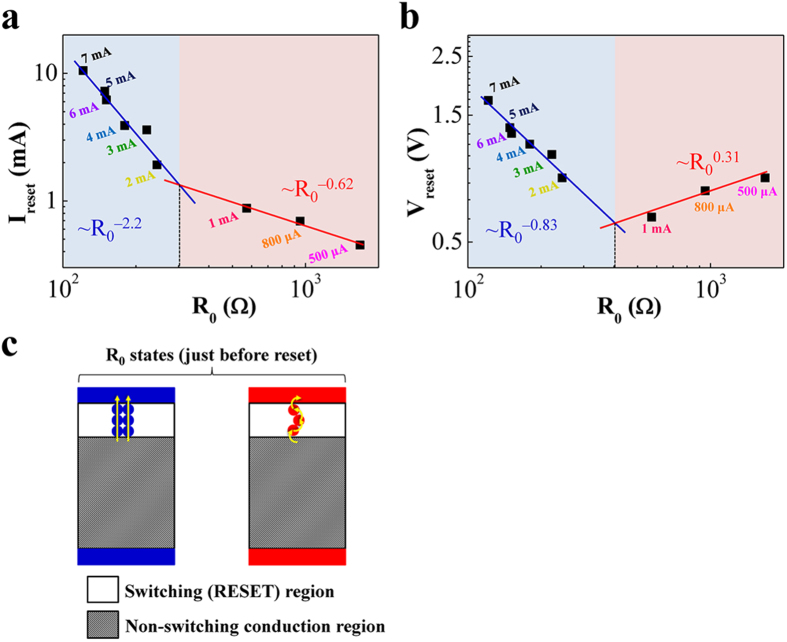
(**a**,**b**) show the relations of I_reset_ vs. R_0_ and V_reset_ vs. R_0_, respectively. Each relation is specifically divided into two characteristic regions according to the exponent x in the proportional term R_0_^–x^. (**c**) The correlation between these scaling behaviors and the topological dimension (or corresponding physical geometry) of the conduction path is depicted.

Figure 2a,b show the relations of I_reset_ vs. R_0_ and V_reset_ vs. R_0_, respectively. Each relation is specifically divided into two characteristic regions according to the exponent x in the proportional term R_0_^–x^. R_0_ was calculated from the linear fit of the I-V curve just before each reset process, as shown in Fig. 1b. Although scaling behavior contains interface-specific information concerning the bottleneck connectivity of the conduction path, the resultant multiple connectivity can be used to validate the geometry-dependent effects against the single connectivity. Thus, we were able to qualitatively determine the filament geometries as a function of reset behavior. The values of the exponent α in the proportional relation I_reset_ ~ R_0_^–α^ were obtained: i.e., 0.62 between 500 μA and 1 mA set-CC and 2.2 between 2 mA and 7 mA set-CC. The values of exponent β in the proportional relation V_reset_ ~ R_0_^–β^ were also measured: i.e., 0.31 between 500 μA and 1 mA set-CC and 0.83 between 2 mA and 7 mA set-CC. The exponents α and β are within error range of values for several metal oxides^19^. Since the resistance criterion (R_C0_) obtained from the cross point of the fitted lines for the 500 μA−1 mA section and the 2–7 mA section can be approximated, the scaling behavior shows two different characteristic relations with respect to R_C0_ = 350 Ω (an average value of approximate 300 Ω for the case of α and 400 Ω for the case of β). R_C0_ of 350 Ω in our Pt/SiO_x_/TiN is larger than the value for several metal oxides (30 Ω) and smaller than the value for a semi-continuous metal (2 kΩ)^18^. The correlation between these scaling behaviors and the topological dimension (or corresponding physical geometry) of the conduction path had been investigated based on the fractal geometry of a percolating cluster^19^. The transport properties through these conducting paths have been experimentally and theoretically correlated to two scaling regimes depending on the number of bottleneck links of conduction paths, as depicted in Fig. 2c 18,19. Using the picture and R_C0_, the conduction path geometry can be extracted. That is, the bottleneck which is directly related to reset switching consists of numerous connections with a large diameter for low resistance values (R_0_ < R_C0_) and very few connections with a small diameter for high resistance values (R_0_ > R_C0_) in the switching region. Interestingly, the two scaling regimes can be indistinguishable in a semi-continuous metal percolation system under the I_reset_ vs. R_0_ scheme. Because the observable change of the exponent α for R_0_ > R_C0_ is explained by hopping conduction via very few connections, the hopping conduction for R_0_ > R_C0_ is distinguished from the more metallic conduction for R_0_ < R_C0_^20^. In this study of a Pt/SiO_x_/TiN system, the obtained R_C0_ value of 350 Ω is larger than the value for several metal oxides (30 Ω) and smaller than the value for a semi-continuous metal (2 kΩ), which can affect the switching behavior caused by thermal effects, compared to that of metal oxides. Moreover, since our Pt/SiO_x_/TiN system basically exhibits BRS and the incorporation of hopping conduction is slightly different from the scaling behavior in URS, the switching characteristics for the two scaling regimes was investigated in more detail.

**Figure 3:**
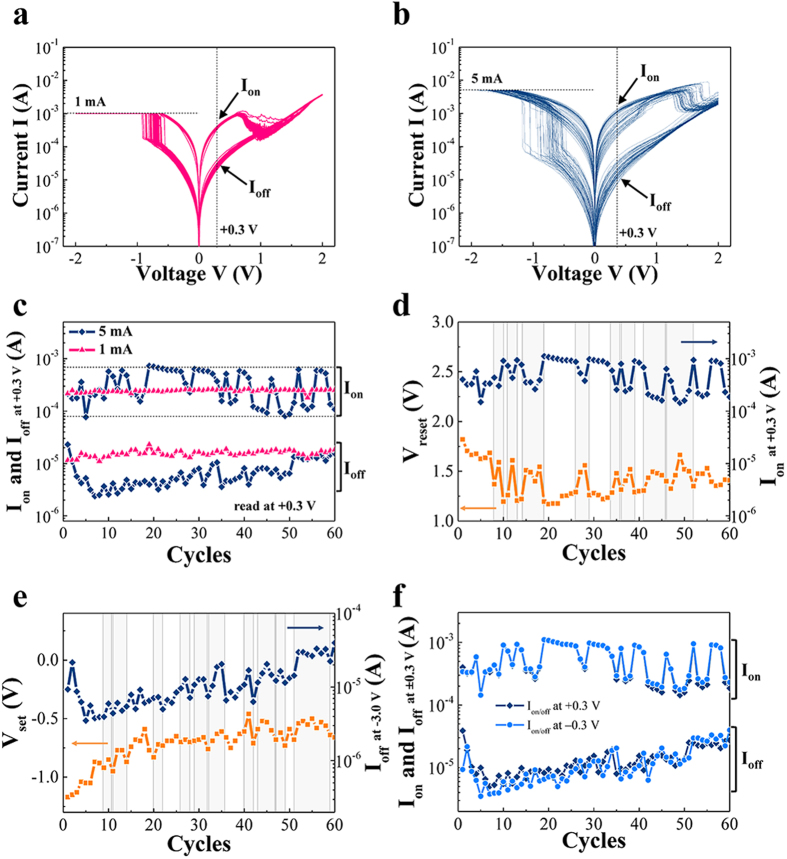
60 switching cycles (a) under 1 mA set-CC (low set-CC) and (b) under 5 mA set-CC (high set-CC). (**c**) Comparison of I_on_ and I_off_ for the low and high set-CC. (measured at +0.3 V) (**d**) Correspondence between V_reset_ and I_on_ for the high set-CC. (**e**) Correspondence between V_set_ and I_off_ for the high set-CC. (**f**) Correspondence of current level with respect to the sign of read voltage is examined.

Among the switching characteristics, the cyclic switching behavior showed a significantly different reproducibility at 1 mA set-CC representing the R_0_ > R_C0_ regime and at 5 mA set-CC, representing the R_0_ < R_C0_ regime. In order to compare the basic cyclic switching behavior for the two scaling regimes, we independently switched the device 60 times under a set-CC of 1 mA (denoted as low set-CC) and 5 mA (denoted as high set-CC) as shown in Fig. 3a,b. The switching behavior under high set-CC conditions was found to be more variable than those under low set-CC: i.e., the I_on_ and I_off_ at a read voltage of +0.3 V under low set-CC are relatively uniform, compared to those under high set-CC in Fig. 3c. The uniform states of switching under low set-CC are definitely determined by the set-CC blocking of the current effectively. In contrast, the current undergoes many fluctuations under high set-CC implying additional memory states, which are indicated by dashed lines in Fig. 3c. To identify the multiple states under high set-CC, correlations among the switching parameters such as V_reset_, V_set_, I_on/off_ at +0.3 V, and I_on/off_ at −0.3 V were examined. Figure 3d shows the correspondence between V_reset_ and I_on_ at +0.3 V. The local maximum for V_reset_ is matched with the local minimum of the I_on_ as indicated by the vertical shadow regions. Similarly, the correspondence between V_set_ and I_off_ at −0.3 V is shown in Fig. 3e. The local maximum point of V_set_ is matched with the local maximum of I_off_ as indicated by the vertical shadow regions. In Fig. 3f, although the I_on_ at +0.3 V and I_off_ at +0.3 V are correlated with I_on_ at −0.3 V and I_off_ at −0.3 V, respectively, I_off_ at +0.3 V and I_on_ at −0.3 V are not correlated with I_on_ at +0.3 V (or V_reset_) and I_off_ at −0.3 V (or V_set_), respectively. In other words, the start of the switching (i.e., switching voltage) depends on the current level immediately before the switching, while the end of switching (i.e., the current level after the switching) appears to be random for both the set and reset processes. Thus, we conclude that I_on_ and I_off_ are independently and randomly determined for the high set-CC.

**Figure 4:**
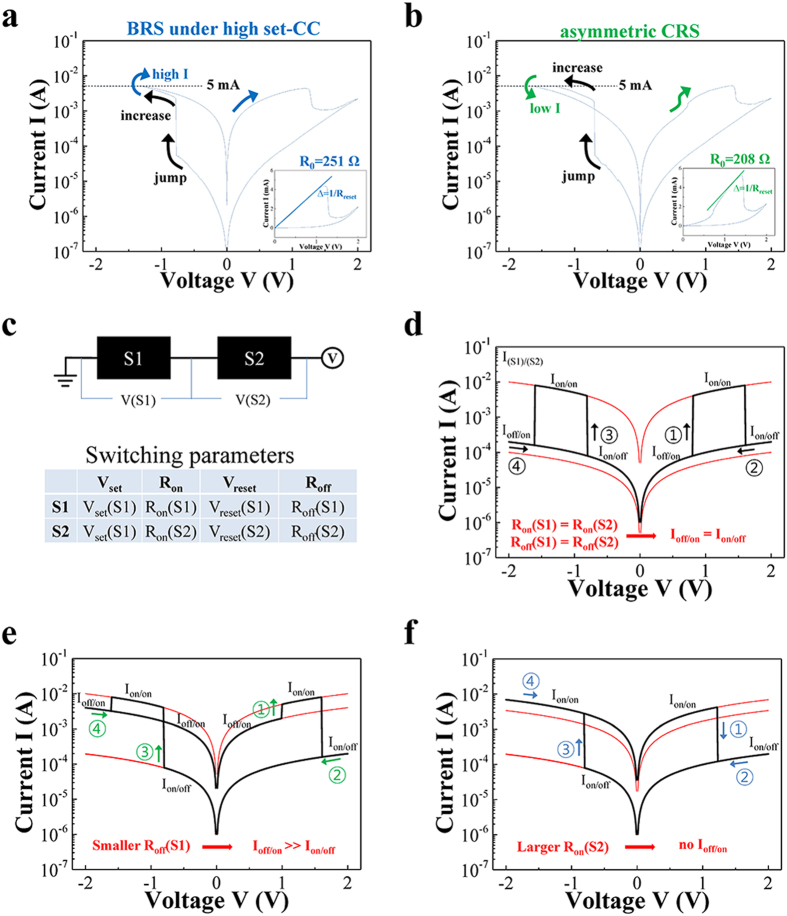
The switching behaviors under the high set-CC can be categorized into (a) BRS behavior and (b) CRS behavior implying anti-serial connection of two BRS devices. (**c**) Double switch model consisting of two resistive switches (S1 and S2) is introduced to determine the origin of the various switching behaviors. (**d**) Typical symmetric CRS was obtained under the exactly same switching parameters of the two switches except the polarity. (**e**) Variation of I-V characteristics which is similar to the one in Figure 4b is generated by lowering off resistances of switch S1. (**f**) Another variation of I-V characteristics which is similar to the one in Figure 4a is generated by the increasing R_on_(S2).

The multiple random states under the high set-CC can be categorized into two characteristic switching modes. One of the switching modes is a denoted ‘BRS under high set-CC’ as shown in Fig. 4a, while the other is a denoted ‘asymmetric CRS’ shown in Fig. 4b. The ‘BRS under high set-CC’ shows a ‘high I’ after a gradual current ‘increase’ following a current ‘jump’ during the set process. On the other hand, the ‘asymmetric CRS’ indicates a ‘low I’ after a gradual ‘increase’ in the current, following a current ‘jump’ during the set process. Because the current ‘jump’ and the following changes to ‘high I’ or ‘low I’ imply double switching, the variations in switching behavior for the high set-CC can be investigated on the basis of a simple model comprised two serial bipolar resistance switches (S1 and S2) with switching parameters, as depicted in Fig. 4c.

Although this model has simple parameters, it is sufficiently useful to permit the double switching behavior to be characterized, owing to the dynamical correlation among the parameters. Although the mechanism of filament modification is rationalized from the reported results, direct consequences of the mechanism cannot uniquely determine the characteristic electrical behaviors presented in this work. Because by excluding other possibilities, the simulation results can reduce the gap between the possible consequences and the actual electrical behavior. For example, the typical symmetric CRS is generated under the voltage sweep when V_set_(S1) = −V_set_(S2), V_reset_(S1) = −V_reset_(S2), R_on_(S1) = R_on_(S2), and R_off_(S1) = R_off_(S2) as shown in Fig. 4d (In general, |V_set_|~|V_reset_| and R_on_ ≪ R_off_ for S1 and S2). Even under the arbitrary parameter values, the relations among the parameters explain the variations of switching behaviors in this experiment sufficiently well. When the R_on_(S1) > R_on_(S2) and R_off_(S1) < R_off_(S2), the typical symmetric CRS varies as shown in Fig. 4e which is similar to the switching behavior in Fig. 4b. It should be noted that a resistance setup for S1 and S2 such that R_off_(S1) + R_on_(S2) ≪ R_on_(S1) + R_off_(S2) (equivalently, I_off_(S1) + I_on_(S2) ≫ I_on_(S1) + I_off_(S2)) is required for the asymmetric CRS, because of R_off_(S1) + R_on_(S2) = R_on_(S1) + R_off_(S2) (equivalently, I_off_(S1) + I_on_(S2) = I_on_(S1) + I_off_(S2)) in Fig. 4d.

In contrast, the switching behavior shown in Fig. 4a would be expected when the S1 remains on-state without reset switching from I_on/on_ to I_off/on_. The S1 can remain on-state in the simulation when the absolute voltage on S1 (i.e., |V(S1)| = |V|∙R_on_(S1)/(R_on_(S1) + R_on_(S2)) for series resistances) is sufficiently small to prevent S1 from satisfying V(S1) < V_reset_ (here V_reset_ < 0 for S1) for reset. Interestingly, the ‘BRS under high set-CC’ shows a higher R_0_ than the ‘asymmetric CRS’, as shown in insets of Fig. 4a,b. Because R_on_(S1) + R_on_(S2) = R_0_, an increase of R_on_(S2) can satisfy both the decreased R_on_(S1)/(R_on_(S1) + R_on_(S2)) for small |V(S1)| and higher R_0_ for the ‘BRS under high set-CC’. Any other resistance modulation situation, such as decreased R_on_(S1), increased R_on_(S1), and decreased R_on_(S2) cannot simultaneously satisfy a decreased R_on_(S1)/(R_on_(S1) + R_on_(S2)) and higher R_0_. A scheme showing possible BRS behavior is represented in Fig. 4f. The requirement of higher resistance for S2 implies a narrower conduction path in respect of geometric shape.

**Figure 5:**
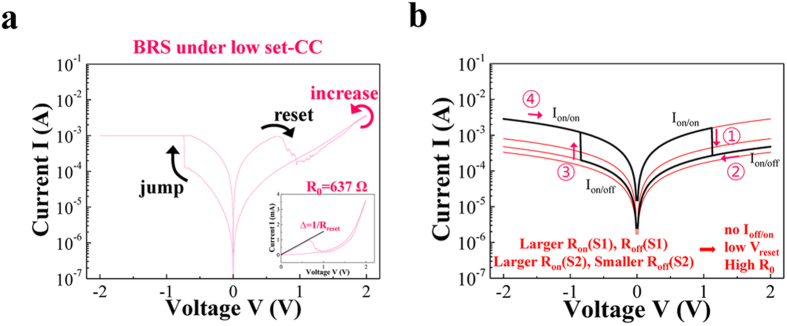
(**a**) BRS under the low set-CC is represented. (**b**) I-V characteristics which is similar to the one in Figure 5a is generated when R_on_(S1), R_off_(S1), and R_on_(S2) are increased except R_off_(S2).

Although the low set-CC is a difficult switching parameter to be defined in the simulation, denoted ‘BRS under low set-CC’ in Fig. 5a can be rationalized from the experimental result which reveals the lowest V_reset_ at 1 mA set-CC in Fig. 1f. From the switching polarity of previous simulation results, ‘jump’ and ‘reset’ are possibly belong to S2 while ‘increase’ after ‘reset’ is possibly belong to S1 in Fig. 5a. The ‘increase’ can be treated as a slight modification of resistance state, because it is not a definite set switching similar to ‘increase’ in Fig. 4a. Thus, following condition, V(S2) for BRS under low-CC > V(S2) for BRS under high-CC, is required for the lower V_reset_ under low set-CC. Since V(S2) = V∙R_on_(S2)/(R_on_(S1) + R_on_(S2)), a decreased R_on_(S1) or an increased R_on_(S2) is required for BRS under low-CC. In addition, the I_off_ for the low set-CC is higher than that for the high set-CC shown in Fig. 3c, while the R_0_ ( = 637 Ω) for the low set-CC is larger than R_0_ ( = 251 Ω, 208 Ω in Fig. 4a,b) for the high set-CC shown in Fig. 5a. Thus, a larger R_on_(S1) + R_on_(S2) and smaller R_on_(S1) + R_off_(S2) are required. Since R_on_(S1) is inconsistently involved in the requirements for a larger R_on_(S1) + R_on_(S2) and a smaller R_on_(S1) + R_off_(S2), a sufficiently larger R_on_(S2) and a sufficiently smaller R_off_(S2) are required regardless of R_on_(S1). A narrower conduction path and then a little deformation of the path are expected due to the larger R_on_(S2) and the smaller R_off_(S2). Interestingly, the reset condition V(S2) ≥ V_reset_(S2) indicates minimum V = V_reset_(S2), because V(S2) = V∙R_on_(S2)/(R_on_(S1) + R_on_(S2)) = a∙V (a ~ 1) under an extremely decreased R_on_(S1) or an extremely increased R_on_(S2). Otherwise, V(S2) = a∙V (a < 1) implies minimum V = V_reset_(S2)/a > V_reset_(S2). The minimum V_reset_ for the 1 mA set-CC implies an extremely decreased R_on_(S1) or an extremely increased R_on_(S2). However an extremely decreased R_on_(S1) is not reasonable to satisfy a larger R_on_(S1) + R_on_(S2). The corresponding simulation result is represented in Fig. 5b.

**Figure 6:**
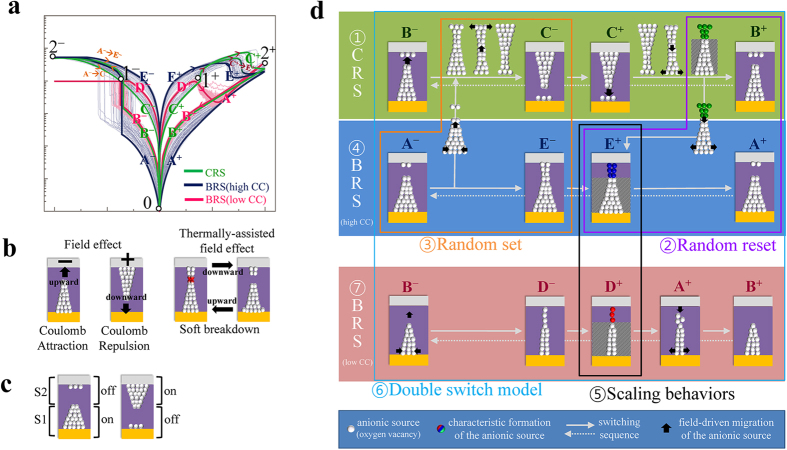
(**a**) The various types of switching for the high set-CC (5 mA) and those for the low set-CC (1 mA) are schematically displayed with particular current levels (letters) and characteristic junctions (numbers). (**b**) Several well-known switching processes such as Coulomb interactions by electric field effect and vacancy relocations by Joule heating effect. (**c**) Proposed oxygen vacancy configuration based on the results from the double switch model. (**d**) Starting with the denoted ①CRS, we can put together whole switching behaviors in the experiments. The diagram is grouped and numbered into some parts according to the subject examined in this experiment.

Because it is known that the metal/oxide and the oxide/metal interface regions play roles of S1 and S2 in metal/oxide/metal CRS devices, double switches can be assigned to the Pt/SiO_x_ and the SiO_x_/TiN interface regions. The V_set_ was typically negative in our Pt/oxide/TiN system exhibiting single level Pt/oxide-interface-mediated switching. Thus, the S1 and the S2 can be assigned to the SiO_x_/TiN and the Pt/SiO_x_ interface regions, respectively. Figure 6a schematically displays the various types of switching for the CRS (green), for the BRS under high set-CC (blue), and for the BRS under low set-CC (red). The letters and numbers are assigned to specific current levels (or corresponding overall resistance) and characteristic junctions. The assigned points and paths in the negative and positive voltage region are marked with superscripts ‘^−^’ and ‘^+^’, respectively. Since Fig. 6b depicts several oxygen-vacancy-mediated (white symbol) switching processes, including electric field-driven switching and thermally-assisted field-driven switching, the combinations of resistance states shown in Fig. 6c and switching processes in Fig. 6b generates a map of switching processes in Fig. 6d according to the sequence of ①−⑦. Figure 6b also starts from the convincing explanation (①CRS) that emerges from various CRS-based experimental results. Furthermore, the schematics not only show previously reported results, but provide new information regarding filament modification as the result of other experimental and simulation findings (②−⑦). ‘①CRS’ denotes oxygen vacancy movement as a function of the voltage sign. The corresponding schematic diagrams B^−^, E^−^, E^+^, and B^+^ are identical to the those reported in other papers^13^. The ‘②Random reset’ notation indicates the random transition from C^+^ to B^+^ or E^+^ to A^+^. The transition from C^+^ to B^+^ can result from field-driven and then thermally-assisted field-driven migration on the basis of CRS in a single layered oxide system, while that from E^+^ to A^+^ would be expected after longer field-driven migration than those for the C^+^ to B^+^ transition. Because the transition from C^+^ to E^+^ indicates longer field-driven migration than the transition from C^+^ to B^+^, the conduction path at the reset process is narrower in ‘BRS under high set-CC’ than that in ‘asymmetric CRS’. It can be rationalized from the double switch model simulation results. Therefore, it can be concluded that the different reset process is dependent on the geometry of the conduction path. ‘③Random set’ also indicates a randomly determined geometric difference such as the transition from A^−^ to C^−^ or E^−^ similar to the ‘②Random reset’. Thus, the ‘④BRS (high CC)’ can be described different from ‘①CRS’. ‘⑤Scaling behaviors’ represents the topologically and geometrically different conduction paths obtained from the relations of I_reset_ vs. R_0_ and V_reset_ vs. R_0_ in Fig. 2. The much narrower conduction path derived from the scaling behaviors is consistent with a larger R_on_(S2) from the double switch model. The ‘⑥Double switch model’ finally describes the various switching behaviors through the switching mediated via the two near-interface regions, as depicted in Fig. 6c. Thus, the ‘⑦BRS (low CC)’ can be described different from ‘①CRS’ and ‘④BRS (high CC)’. Finally, connecting each switching process, we can combine the collection of switching behaviors in the experiments, as shown in Fig. 6d. Therefore, we conclude that the co-occurrence of ‘asymmetric CRS’ and ‘BRS under high set-CC’ are due to different conduction path geometries even under the same set-CC.

**Figure 7:**
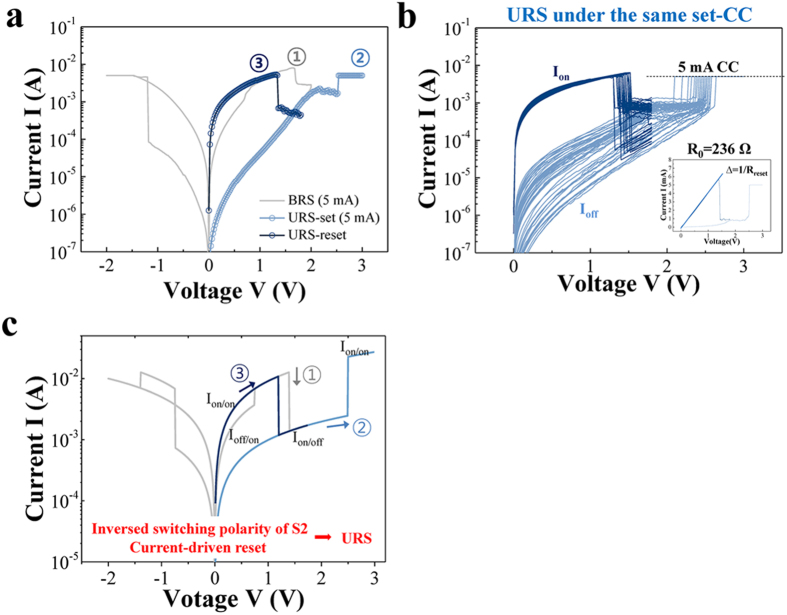
(**a**) URS can be induced by increasing the voltage after ①reset of BRS under set-CC of 5 mA. The ②URS set was induced under set-CC of 5 mA (the same as BRS) and ③URS reset was induced without current compliance. (**b**) 60 switching cycles for URS under the 5 mA set-CC. (**c**) Double switch model explains the URS as the inversed polarity of the switch S2 in high voltage and the current-driven reset.

Interestingly, URS was directly induced after the asymmetric CRS as shown in Fig. 7a. Under the 5 mA set-CC, the URS set process was observed in a voltage sweep from 0 to +3. A voltage sweep from 0 to +1.8 V without current compliance then induced a URS reset. The cyclic URS behaviors show less dispersive I_on_ and more dispersive I_off_ in Fig. 7b compared to the BRS (including the CRS). This different dispersion of on/off states between BRS (including the CRS) and URS under the same set-CC for 5 mA indicates the existence of a different switching mechanism that originates, not from the current but the geometry of the conduction path. The conduction path can be narrower than those of the BRS and the CRS under the same high set-CC at the top electrode, because the URS set is a similar to forming-off process, i.e., a current jump under the Coulomb repulsion^11^,^21^. The current jump under the Coulomb repulsion possibly generate lower oxygen vacancy concentration at the metal/oxide interface than that under the Coulomb attraction. Therefore, an inversed upright conical conduction path would be formed. Which is possibly related to the drastic decrease of I_off_ for the URS due to thermal effect. Using a double switch model, we conclude that the occurrence of the URS can be attributed to the inverse polarity of the switch S2 at a high voltage, as shown in Fig. 7c. Because the inversion of polarity is changed during the first URS set process in the model, the reset of S2 must be triggered by the current and not the voltage. Thus, the use of the double switch model results in the conclusion that URS is thermally-driven switching, as is widely known. The corresponding simulation result is represented in Fig. 7c.

**Figure 8:**
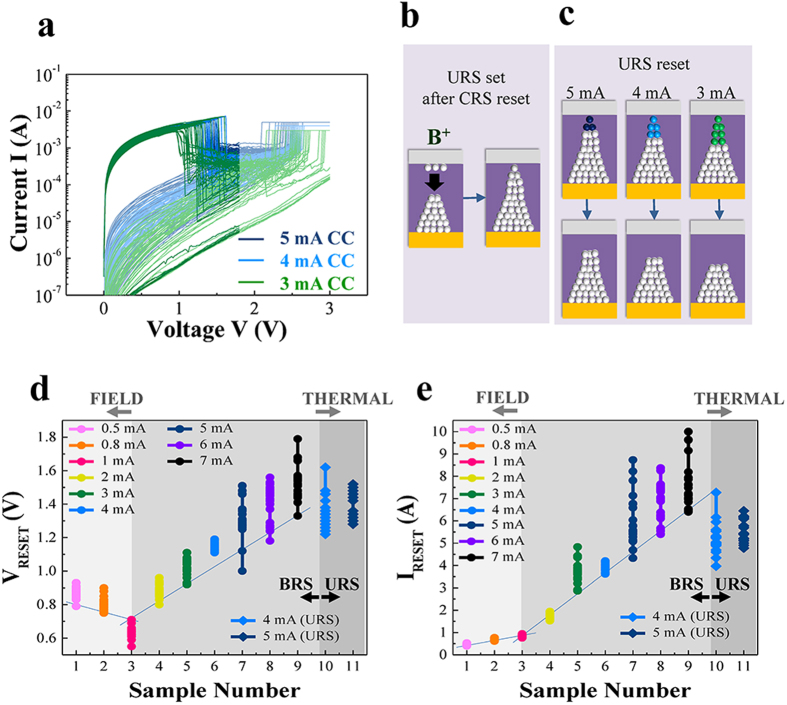
(**a**) The URS behaviors under 3, 4, and 5 mA set-CC were compared. (**b**) Schematic diagrams for the inducement of URS after CRS reset under the same 5 mA set-CC. (**c**) Proposed URS set and reset are described with respect to the current compliance. (**d**) V_reset_ and (**e**) I_reset_ are displayed and they show characteristic dependence on the set-CC.

To investigate the different oxygen vacancy configurations after the URS set, the URS behaviors under different set-CC conditions were compared in Fig. 8a. The URS under 3−5 mA set-CC in Fig. 8a shows a decreasing V_reset_ with decreasing set-CC. Moreover, the current with the V_reset_ was decreased to a greater extent. That is, the lower set-CC condition induces a more resistive off-state, implying a longer oxygen vacancy depletion region after reset, which can be explained by a narrower conduction path due to a lower CC and consequently, that the thermal effect has a considerable influence on the process, due to the narrower conduction path. Since the set process for URS under 5 mA CC was induced by an increased voltage immediately after the reset of CRS under 5 mA CC, this implies the downward movement of oxygen vacancies due to Coulomb repulsion in addition to the vacancy generation^11^,^21^, as shown in Fig. 8b. The conduction path geometry is an inversed upright conical shape. Also, the absence of CRS-like behavior after the transition to URS assures a definite conduction path near the bottom electrode. The different filament dimensions determined by the set-CC were compared and the results are shown in Fig. 8c. The differently colored symbols indicate a region where the Joule heating effect is dependent on the dimension of the conduction path; the narrower is the conduction path, the stronger is the Joule heating effect. The great differences in the I_off_ in BRS and URS provide support for the oxygen vacancy distributions depicted in the schematics from the results where the I_off_ of URS is much lower than that of BRS under 5 mA CC and the I_off_ of URS under 3 mA and 4 mA CC is much lower than that of the URS under 4 mA and 5 mA, respectively.

The dominance of the field or thermal effects can be clearly distinguished by examining the distribution of I_reset_ and V_reset_. The V_reset_ distribution had the lowest value at the 1 mA CC condition and the I_reset_ value steeply increases thereafter, as shown in Fig. 8d,e, respectively. Considering the V_reset_ and I_reset_, resistive switching under various set-CC conditions can be categorized as field-driven, thermally-assisted field-driven, and thermally-driven switching. That is, a low-CC requires a high V_reset_ in field-driven switching to achieve oxygen vacancy movement due to its low concentration. In thermally-driven switching, the I_reset_ was high and its distribution was less uniform than that in field effect dominant conditions, which can be attributed to non-directional thermal dissolution of the conduction path. In the case of intermediate thermally-assisted field-driven switching, the I_reset_ increased with increasing CC but the distribution of I_reset_ was not much more dispersive than that in thermally-driven switching. Although the degree of significance of the thermal effect arising from a high operation current is difficult to determine, the entangled co-occurrence of three different switching modes in SiO_x_ can be resolved. In contrast to several metal oxides in which the BRS related to the field effect and the URS related to the thermal effect are discretely understood due to the great difference in operation current levels, it should be noted that the early occurrence of the thermal effect in SiO_x_ indicates the different origin of the conduction path.

**Figure 9:**
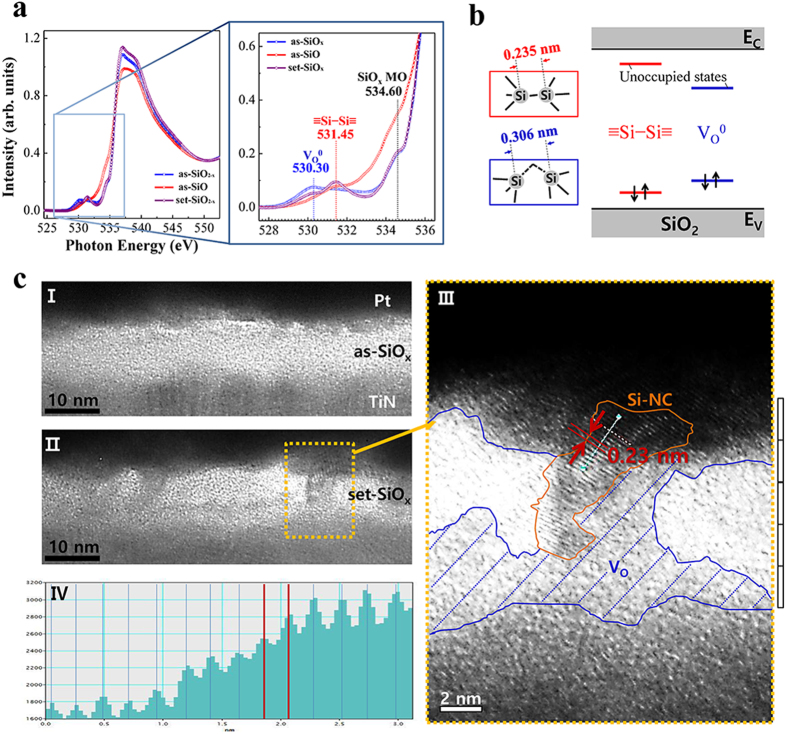
(**a**) O-K edge X-ray absorption spectra for as-SiO_x_ (blue line), as-SiO (red line), and set-SiO_x_ (purple line) subjected to a set-CC of 7 mA. (**b**) The defect levels arising from Si−Si nano-crystalline or oxygen vacancy. The left-side indicates the length between Si atoms in the defect in the different defect states. **(c**) High-resolution TEM images for (I) as-SiO_x_, (II) set-SiO_x_, (III) the region indicated by the dashed rectangle in region II, and (IV) lattice spacing along the nano-crystalline in region III. The red lines delineate the lattice spacing (2.965/13 ≈ 0.23 nm) in region III.

In order to investigate the source of the change in resistance, we performed XAS using a synchrotron beam. Since the change in the elemental components of the conduction path in silicon oxides is related to oxygen deficiency, the XAS characteristics between the pristine SiO_x_/TiN (labeled as as-SiO_x_) and the BRS set-operated SiO_x_/TiN under 7 mA CC (labeled as set-SiO_x_), the highest CC utilized in this study, were compared. The O-K (oxygen K-shell) edge absorption spectra shown in Fig. 9a provide information regarding changes in the pre-edge peaks at 530.30 eV and 531.45 eV in front of the absorption edge with a shoulder peak of the a-SiO_x_ molecular orbital (MO) state at 534.60 eV^22^, where the pre-edge peaks at 530.30 eV and 531.45 eV were remarkable in both the as-SiO_x_ and the as-SiO. The one at 530.30 eV can be assigned to an oxygen vacancy while the other at 531.45 eV can be assigned to nano-crystalline Si-Si (NC). The peak assignment can be rationalized based on previously reported theoretical and experimental results^8^,^23^. The DFT calculations predict that the origins of unoccupied states are (strong) Si-Si bonds and an oxygen vacancy (or weak strong Si-Si bond) as oxygen-deficient defect states^23^. The Si-Si bond in amorphous SiO_2_ can be ‘locally’ possible, although it is unlikely that the entire SiO_2_ would become Si. Si-NCs can be formed in a SiO_2_ matrix under conditions of high temperature annealing^24^,^25^. The Si DB can be a transition region between Si-NCs and the SiO_2_ matrix^26^. Under conditions of RRAM operation, the electrical stress is sufficiently strong to break Si-O bonds and temperature can become quite high due to the high current density^27^. In addition, the 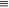
Si−Si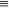 bond was closer to the absorption edge than the oxygen vacancy in the calculation as shown in Fig. 9b, although the exact defect levels could not be predicted. In particular, X-ray photoelectron spectroscopy (XPS) and Electron spin resonance (ESR) measurements determined the most probable oxygen-deficient defect state of •Si
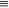
Si_3_ dangling bonds in the normal (SiO_x < 0.8_) URS^8^. Because the URS in this study exhibited normal URS behavior and both the Si−Si bond and the oxygen vacancy originate from •Si
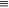
Si_3_ dangling bonds, it is likely that the Si−Si NCs are the origin of elemental change in our Pt/SiO_x_/TiN switching device. Interestingly, the peak related to an oxygen vacancy was decreased, while the peak related to the Si−Si NC was increased in the set-SiO_x_ in Fig. 9a. In stoichiometric a-SiO_2_, two silicon atoms are connected by one bridging oxygen atom. When the oxygen atom is removed, the two neighboring silicon atoms establish common bonds, which are the origin of the oxygen vacancies or Si−Si bonds. The left of Fig. 9b depicts the bond length and the right indicates possible corresponding gap-states. A shorter bond length corresponds to larger splitting of gap-states and a defect state would be expected to be closer to the absorption edge in XAS. The bond lengths of 0.235 nm and 0.306 nm indicate Si-Si bonds in crystalline bulk silicon and the possible loose Si-Si bond (i.e., oxygen vacancy) length in a-SiO_2_, respectively^23^,^28^. In general, since the defects are embedded in an amorphous SiO_2_ host material, the characteristic bond length can be distributed between 0.23 and 0.27 nm^29^. Although it is uncertain whether the Si-NCs in this experiment can be caused by a Si-Si bond in crystalline Si or a Si-Si bond in a-SiO_2_, the shorter Si-Si bond length (0.235 nm or 0.23 ~ 0.27 nm) than the weak Si-Si bond length (0.306 nm) is consistent with the relative position of the defect states.

The oxygen-vacancy-formed Pt/SiO_x_/TiN and pristine Pt/SiO_x_/TiN structures were confirmed *via* high-resolution transmission electron microscopy (HRTEM), as shown in Fig. 9c. The TEM image of the pristine Pt/SiO_x_/TiN structure shows that the 10-nm-thick SiO_x_ amorphous structure was deposited on crystalline TiN, as shown in Fig. 9c-I. Figure 9c-II shows a cross-sectional image of the set-operated Pt/SiO_x_/TiN structure after several operations under 7 mA set-CC. The image shows that crystallization is locally generated in the SiO_x_ layer. The magnified HRTEM image shown in Fig. 9c-III provides clear evidence of the presence of a NC region in the SiO_x_ layer and the lattice spacing was determined to be approximately 0.23 nm (≈2.965/13) in the region of the NC structure using line profiling of the image contrast along the light-green line, as shown in Fig. 9c-III,IV. The lattice spacing of ~0.23 nm corresponds to a Si−Si bond in the (211) plane. Thus, the results of the XAS and HRTEM investigations indicate that broken Si−O bonds are generated and Si NC conduction paths are formed under the on state of the SiO_x_ layer. The experimental results from XAS (the presence of two characteristic peaks) and TEM (the coexistence of crystalline and non-crystalline contrasting area) actually showed that the filamentary path is comprised of Si-NCs and oxygen vacancies. Therefore, we concluded that the oxygen vacancy mainly modulated the filament and that Si-NCs were additionally formed due to the high current density. Although we did not observe a clear change in the conduction path under the switching process with a lower CC, the results showing the generation of Si−Si NCs and pre-edge peaks strongly support the formation of a conduction path of Si−Si NCs under the switching process. This result is in contrast to resistive switching, which occurs via the migration of electrode Pt, i.e., a more resistive conduction path (Si−Si NCs) rather than a metallic conduction path (Pt) is well supported by the early occurrence of a thermal effect at a low CC, as discussed previously.

**Figure 10:**
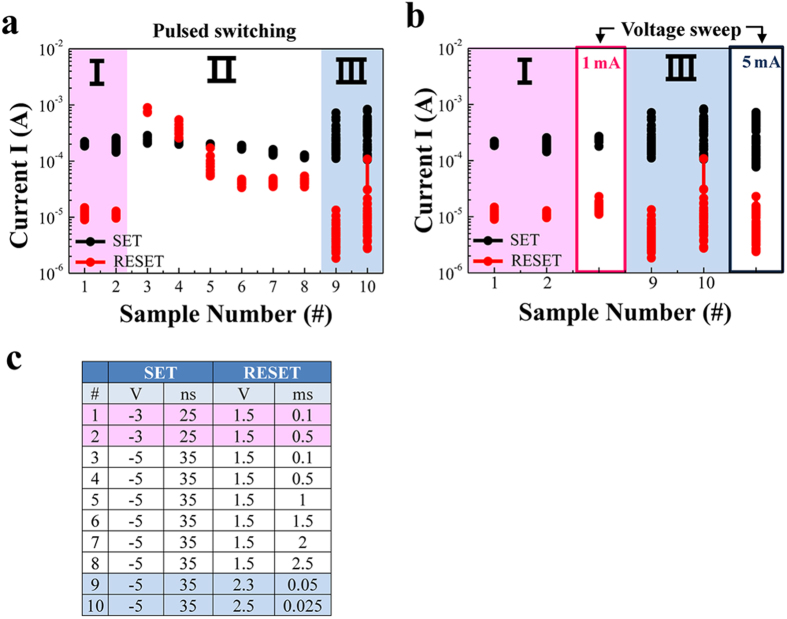
Pulse-dependent characteristics of the set/reset states according to the mechanism of the reset process. (**a**) Memory cycles according to various set/reset conditions read at +0.3 V for sample numbers (SN) 1 to 10. (**b**) The results of the pulsed switching compared with those of voltage sweeping. (**c**) A table showing the pulse conditions according to the SN. Symbols I, II, and III are used to distinguish the results.

**Figure 11:**
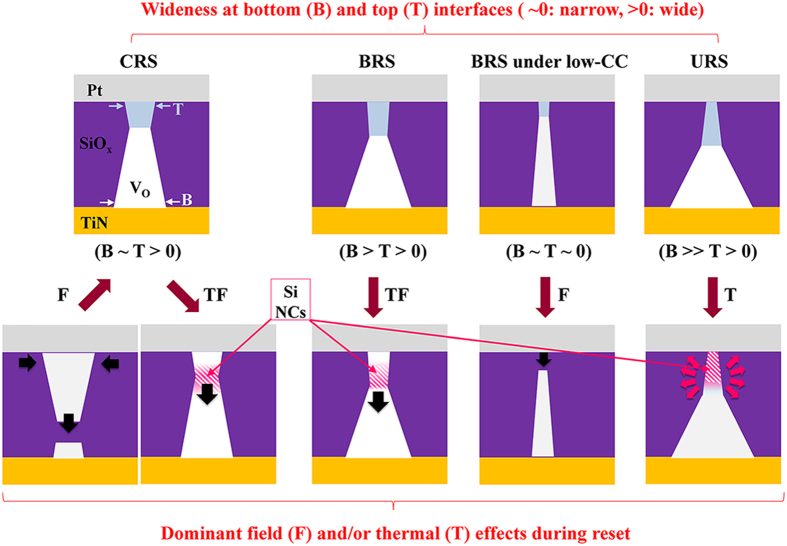
Filament geometry induced electric field effect and/or thermal effects are summarized.

Cyclical testing was carried out using various electric pulses in order to investigate the differences in the switching process based on the field and thermal effects in terms of the height and width of the pulse. Figure 10a shows I_on_ after set pulse and I_off_ after reset pulse at a read voltage of +0.3 V after each pulse switching. The conditions of the pulses are summarized in the table shown in Fig. 10c. Two types of pulses were used to induce the effects of the high- and low-set CC related to thermally-assisted field-driven and field dominating switching: a 25 ns pulse of −3 V (sample number (SN) 1 and 2) and a 35 ns pulse of −5 V (SN from 3 to 10) for the set operation, respectively. Figure 10a shows the three distinct regions, namely I, II, and III, showing relatively uniform, improper (inversed states or narrow memory states), and dispersive respective I_on_ and I_off_ values. We compared the I_on_ and I_off_ values and their dispersions in regions I and III with those for BRS obtained under 1 mA and 5 mA CC conditions, as shown in Fig. 10b. In region I, the I_on_ and I_off_ values and their consistency are similar to those for BRS under 1 mA CC. In region III, the I_on_ and I_off_ values and their inconsistency are similar to those for BRS obtained under 5 mA CC. Thus, the conditions of the set pulses for regions I and III correspond to low set-CC and high set-CC, respectively. In addition, the conditions of the reset pulses show similar characteristic results as that for voltage sweep switching: 1) the gradual (under low set-CC in the inset of Fig. 5a) and abrupt (under high set-CC in the insets of Fig. 4a,b) reset in voltage sweep switching are related to the wide and narrow widths of the pulses in pulsed switching and 2) the increased pulse width with a constant low voltage, which induces inversed on/off states, corresponds to the CRS under an insufficiently increased voltage sweep (transition from I_off/on_ to I_on/on_ in Fig. 4e), as shown in Fig. 10a-II. A more increased pulse width induces normal (BRS) on/off states from the CRS. These results indicate that a both a specific length of time and a high voltage are needed for field-driven switching and thermally-assisted field-driven switching, respectively. In summary, the schematic diagram of the various reset processes for BRS under low set-CC, BRS (under high set-CC), CRS (under the same high set-CC), and URS (under the same high set-CC) can be classified by different conduction path geometries as depicted in Fig. 11. For example, ‘B ~ T > 0’, which means sufficiently and compatibly wide bottom (B) and top (T) conduction path, distinguishes the CRS from other switching behaviors. Thus, thermally-assisted field-driven (TF) migration can be the driving force for reset in CRS. ‘B ~ T’ implies that the mirrored conduction path geometry is formed under the opposite voltage sign. Similarly, BRS is a thermally-assisted field-driven switching due to less-wide top and wider bottom conduction paths. The switching region is near the top electrode in contrast to CRS. BRS under low-CC is a field-driven switching due to narrow top and bottom conduction paths. URS is a thermally-driven switching due to the opposite directional top conical and much wider bottom conduction paths.

## Conclusions

We investigated three different switching modes, namely, bipolar, complementary, and unipolar resistive switching (BRS, CRS, and URS, respectively), in a single Pt/SiO_x_/TiN stack. Although the transition from BRS to URS is typically irreversible due to the great difference in operating current level, the transition from BRS to URS in our Pt/SiO_x_/TiN structure was observed at the same set-CC. Based on the geometrical evolution of the filamentary path in single oxide complementary resistive switching, the unusual co-occurrence of three different switching modes at the same set-CC is explained as filament-geometry-dependent electric field and/or thermal effects. In contrast to the role of oxygen vacancies in metal oxides, nano-crystalline Si–Si bonds in a-SiO_x_ can result in the unusual co-occurrence. For the best memory performance, the method used to determine the suitable degree of thermal effect to optimize the reproducibility, switching speed, and power consumption of switching should be solved beforehand. The compliance-based analysis of the voltage-sweep resistive switching and the corresponding pulsed switching in SiO_x_ conducted in this study can be helpful in our understanding of field and thermal effects in resistive switching. The results provide a typical example of how to effectively optimize device operation by controlling the geometry of conduction path.

## Additional Information

**How to cite this article**: Lim, D.-H. *et al.* Filament Geometry Induced Bipolar, Complementary, and Unipolar Resistive Switching under the Same Set Current Compliance in Pt/SiO_x_/TiN. *Sci. Rep.* **5**, 15374; doi: 10.1038/srep15374 (2015).
